# Baculovirus displaying SARS-CoV-2 spike RBD promotes neutralizing antibody production in a mouse model

**DOI:** 10.1186/s43141-023-00472-2

**Published:** 2023-02-09

**Authors:** Mohamed A. Wahba, Dina Mofed, Doaa A. Ghareeb, Jihad I. Omran, Tamer Z. Salem

**Affiliations:** 1 Molecular Biology and Virology lab, Biomedical Sciences program, UST, Zewail City of Science and Technology, October Gardens, 6th of October City, Giza 12578 Egypt; 2 Bio-screening and preclinical trial lab, Biochemistry Department, Faculty of Science, Alexandria University, P.O. Box 21511, Alexandria, Egypt; 3 Department of Microbial Genetics, Agricultural Genetic Engineering Research Institute (AGERl), ARC, Giza, 12619 Egypt; 4 National Biotechnology Network of Expertise (NBNE), Academy of Science Research and Technology (ASRT), Cairo, 11334 Egypt

**Keywords:** SARS CoV-2, Pseudotyped virus, Baculovirus, Neutralizing antibody, AcMNPV, RBD, Spike

## Abstract

**Background:**

There is always a need for a safe and efficient vaccine platform, especially when facing a pandemic such as COVID-19. Most of the SARS-CoV-2-based vaccines are based on the full spike protein, which is presented as a trimerized protein, and many viral vector vaccines express the spike protein into the host cells and do not display it on virus surfaces. However, the spike receptor-binding domain (RBD)-based vaccines are efficient and are currently under investigation and clinical trials.

**Methodology:**

In this study, we are testing the efficacy of the RBD displayed on a baculovirus as a mean to formulate a safe and stable carrier to induce the immune system against SARS-CoV-2. Therefore, two pseudotyped baculoviruses were constructed to display the RBD, AcRBD-sfGFP-64, and AcRBD-sfGFP-V, using two different displaying strategies based on gp64 and VSV-G envelope glycoproteins, from *Autographa californica* multiple nucleopolyhedrovirus (AcMNPV) and vesicular stomatitis virus (VSV), respectively. BALB/C mice were immunized with the pseudotyped baculoviruses in a dose-optimized manner. Dot blot and Western blot were used to screen and validate the polyclonal antibodies’ specificity to the SARS-CoV-2 RBD. A plaque reduction neutralization test (PRNT) was used to measure the sera neutralization capacity against a SARS-CoV-2 wild-type isolate from Egypt. ELISA was used to quantify certain cytokines for the assessment of the immune response.

**Result:**

The outcome of our investigation showed that the monomeric RBD proteins were properly displayed on baculovirus and efficiently triggered the mouse immune system. The produced sera efficiently neutralized about 50% of SARS-CoV-2 in more than 100-fold serum dilution. The immunized mice showed a significant increase (*p*<0.01) in the levels of IL-2 and IFN-γ and a significant decrease (*p*<0.01) and (*p*<0.001) in the levels of IL-4 and IL-10, respectively, which suggest that AcRBD-sfGFP-64 and AcRBD-sfGFP-V induce Th1 cellular immune response.

**Conclusion:**

The produced recombinant viruses can induce the immune response without adjuvant, which needs dose optimization and further stability tests. Neutralizing antibodies were induced without affecting the health of immunized mice. Th1 response can be attainable through the system, which is of great benefit in SARS CoV-2 infection and the system can be tested for future applications including vaccine development and polyclonal antibody production.

**Supplementary Information:**

The online version contains supplementary material available at 10.1186/s43141-023-00472-2.

## Background

Emerging infectious disease refers to the recent appearance of unknown or previously known pathogens [1]. Emerging viruses can be dangerous, especially with the lack of the necessary equipment and facilities. The lack of biosafety-level facilities in many developing countries has a negative impact on studies related to emerging viruses [2]. Moreover, the need for biocontainment infrastructure to produce live-attenuated or inactivated vaccines imposes a major limitation [3]. An alternative safe platform that can be adaptable and versatile is highly needed [3]. The current pandemic of COVID-19 showed us how low-income countries suffered greatly during this crisis as 15.9% of their populations received at least a single dose compared to 65.5% of the total worldwide vaccination [4]. Baculovirus may hold significant potential for the current and future pandemics in terms of safety, immunogenicity, and its versatility to be tailored against various pathogens. Baculovirus is an insect virus that has been recognized as a non-human viral vector. The double-stranded DNA *Autographa californica* multiple nucleopolyhedrovirus (AcMNPV) is the prototype of baculoviruses that has been used extensively to produce eukaryotic complex proteins [5]. AcMNPV poses many advantages over other viral vectors, including the ease of production with a high titer, the ability to simultaneously deliver multiple genes, the transduction ability to many mammalian cells, and its non-pathogenicity non-replicative nature in humans [6–8]. The primary envelope glycoprotein gp64 is the most abundant envelope protein and is essential for both insect cell infection and mammalian cell transduction [9]. Many researchers have been able to display many proteins such as glutathione-S-transferase, HIV GP120 protein, rubella virus envelope protein, and synthetic IgG-binding domains on the baculovirus membrane [10–12]. On the other hand, VSV-G and HA proteins can be displayed on baculovirus independently from gp64 protein [11, 13, 14]. Usually, the display sequences are derived from gp64 and VSV-G from three main components: the signal sequence, the transmembrane domain, and the cytoplasmic domain [15]. Indeed, baculovirus is an excellent immunogen, as it triggers proinflammatory cytokines via the TLR9 pathway. This inherent property is due to a highly abundant structurally distinct unmethylated CpG motif in its DNA [16]. The adjuvant property plays a major role in dendritic cell maturation, followed by the induction of both cellular and humoral immune responses [15, 17]. Moreover, mammalian promoters such as the CMV promoter or the display by other membrane fusion proteins like VSV-G or human endogenous retrovirus (HERV) envelope protein can enhance transduction efficacy and immune response [18–20]. Nevertheless, the displayed antigen on baculoviral will not necessarily require such modifications, as it will confer potent humoral and cellular immunity upon administration [17].

The beta coronavirus family encompasses the causative agent of COVID-19 severe acute respiratory syndrome virus (SARS CoV-2) along with the Middle East respiratory syndrome virus (MERS-CoV) and severe acute respiratory syndrome virus (SARS-CoV) [21, 22]. SARS CoV-2 is a single-stranded RNA virus (~30 kb) with four structural proteins membrane (M), envelope (E), spike (S), and nucleocapsid (N) proteins [23]. The ACE2 binding and fusion events are organized by the S protein. It has two main subunits the S1 and S2 that are cleaved during its biosynthesis by the host cell furin proteases. The virus binding is catalyzed by the S1 subunit when interacting with host cell receptor ACE2 through the receptor-binding domain (RBD) followed by the fusion with the cell membrane via the S2 subunit fusion peptide [23, 24]. Currently, most of the vaccine arsenal depends on the full S protein. However, the vaccine design based on the RBD may offer a better safety profile. Several studies reported abnormal protein splicing, and alternative polyadenylation (APA) events of the Spike protein were associated with adenovirus vector vaccine (ChAdOx1 nCoV-19) in vitro. The mechanism may lead to C-terminal truncation producing soluble spike protein and binding to endothelial cells via the ACE2 receptor, which might be the cause of thrombosis after an antibody attack to such a complex [25–28]. Therefore, vaccines based on the RBD, the most targeted region by the neutralizing antibodies will be of great value to the current pandemic [29]. The platform and the adjuvant other than the immunogen may shape the immune response differently. For instance, subunit vaccines may shift the balance of Th1/Th2 CD4+ T cell response [28, 30]. In addition, the currently most used vaccines such as mRNAs can induce more neutralizing antibodies while the adenovirus vector vaccine can promote specific cytotoxic T cells efficiently [31].

Researchers have put tremendous efforts to develop various RBD-based vaccines that are already in the preclinical and clinical trials. These vaccines are adopted in several systems and have different immune responses (summarized in supplementary data, Table 1). For example, BNT162b1 and ArCov carry SARS-CoV-2 RBD mRNA triggering the production of CD4+ and CD8+ cells and increasing the production of IFN-γ and IL-2, resulting in enhancing Th1 immune response [32, 33]. Folded RBD-PreS fusion vaccine is based on the fusion of SARS-CoV2 “RBD with hepatitis B virus (HBV) antigen in N and C terminus and expressed in *Escherichia coli* expression system, which promotes the production of allergen-specific IgG responses (IgG1 and IgG 4) [34]. Also, the ZF2001 vaccine is an adjuvant based-protein subunit vaccine, which promotes Th1 cytokine production (IFN-γ, IL-2) and Th2 cytokine production (IL-4), resulting in increasing Th1 and Th2 immune responses [35, 36]. A yeast-expressed RBD-based SARS-CoV-2 vaccine triggers the responses of CD4+ and CD8+ T cells resulting in the promotion of the Th1 cellular immune response [37]. Moreover, west china hospital has developed a recombinant RBD of SARS-CoV2 `spike protein expressed in the baculovirus expression system. It induces the production of IgM and IgG antibodies and enhances the levels of IFN-γ and IL-4 from isolated lymphocytes, resulting in promoting Th1 and Th2 immune responses [38]. Moreover, Song and his colleagues have improved the mutant RBD vaccine expressed in the HIV-1 backbone, which induces the production of highly potent neutralizing antibodies against SARS-CoV-2 WT strain or their variants including Beta, Delta, Alpha, Iota, Kappa, or A.23.1 [39]. We considered the above-stated facts to ensure a safe vaccine platform of baculovirus that has no previous immunity in humans. This can be a useful tool with the increasing evidence of autoimmune disorders associated with COVID-19 and some of its vaccines [40].

In this work, we have selected baculovirus as a safe and efficient platform to display the monomeric spike receptor-binding domain (RBD) fused with a superfolder green fluorescent protein (RBD-sfGFP) as a tracking marker for the produced pseudovirus particles. The RBD-sfGFP fused protein was previously used in another study, and its binding capacity to ACE2 was already confirmed; hence, the 3D structure and function of the RBD are not affected by the sfGFP fusion [41]. The immunogenicity of the produced pseudotyped baculoviruses was assessed and discussed. The obtained sera showed neutralizing activity against a wild-type SARS CoV-2 with elevated Th1 response.

## Methods

### Cell lines

The Spodoptera frugiperda-Sf9 insect cell line (ATCC) was maintained in a monolayer at 26°C with an ExCell-420 insect medium (Sigma Aldrich, USA) supplemented with 10% fetal bovine serum (FBS) (Gibco, ThermoFisher Scientific, USA).

### SARS-CoV-2 RBD-sfGFP pseudotyped baculoviruses production

To produce SARS-CoV-2 pseudotyped baculoviruses, AcRBD-sfGFP-64 and AcRBD-sfGFP-V, the RBD-sfGFP fragment was cloned between gp64 mature domain (TM+CTD) and gp64 signal sequence or VSV-G mature domain (TM+CTD) and VSV-G signal, respectively. Gp64 and VSV-G sequences were PCR amplified from AcMNPV genomic DNA and VSV-G from pCMV-VSV-G, a gift from Bob Weinberg (Addgene plasmid # 8454; http://n2t.net/addgene:8454; RRID: Addgene 8454), respectively. The gp64 and VSV-G signal sequences were cloned into pFastBac™ Dual vector using *Bam*HI–HF and *Eco*RI-HF. The mature domain of gp64 and VSV-G sequences were cloned in the same vector using *Xba*I and *Hind*III-HF. The RBD-sfGFP DNA fragment was PCR amplified from pcDNA3-SARS-CoV-2-S-RBD-sfGFP, a gift from Erik Procko (Addgene plasmid # 141184; http://n2t.net/addgene:141184; RRID: Addgene141184) and cloned using *Eco*RI-HF and *Xba*I between the signal sequence and mature domain of the gp64 and VSV-G. The recombinant vectors were authenticated by sequencing (supplementary. In addition, AcEGFP was previously constructed and used in this study as a control virus [42]. Another control baculovirus (AcDH10Bac) was prepared by transfecting Sf9 cells with DNA isolated from DH10Bac using Cellfectin II reagent (ThermoFisher, USA). The produced virus stocks of AcRBD-sfGFP-64, AcRBD-sfGFP-V, AcEGFP, and the control virus were purified by the Pierce™ Strong Anion Exchange Spin Column, Mini (ThermoFisher, USA).

### Virus quantification and verification by endpoint dilution, in-gel fluorescence, and transmission electron microscope

Virus stocks were tittered by the end-point dilution. In 96 well-plate, Sf9 cells were seeded at a density of 2×10^4^ cells/100μl. A 10-fold serial dilution of the virus stock was made from 10^-2^ to 10^-9^ and each dilution was plated in 12-well replicas. After seven days of infection, the infected wells were calculated by fluorescent microscope using a FITC filter (Olympus, Japan) [43]. In-gel fluorescence was used to confirm the fluorescent activity of the fused RBD-sfGFP protein, as described in [44]. For further confirmation and identification, transmission electron microscopy (TEM) was used to identify the structure of the purified AcRBD-sfGFP-64 (10^8^ pfu/ml) in the electron microscopy unit at Mansoura University. The purified AcRBD-sfGFP-64 (10^8^ pfu/ml suspension) was loaded on a carbon-coated Cu-grid (200 mesh) for 5 min at RT and negatively stained with 2% phosphotungstic acid for 3 min before air drying on a filter paper. The samples were examined using a transmission electron microscope (Jem -2100, USA) at 200 KeV.

### In vivo experiment

The mice were treated in accordance with the guidelines and policies of the National Institute of Health (NIH) animal care. All experiments were approved by the research ethics committee (IACUC#50-2S-1121) issued from the Pharmaceutical & Fermentation industries Development center, SRTA-City. Twenty female BALB/C from 4- to 6-week-old mice were purchased from the National Research Center (NRC). The mice were kept in universal polypropylene cages and divided into four different groups for injection: AcRBD-sfGFP-64, AcRBD-sfGFP-V, AcEGFP, and phosphate-buffered saline (PBS) buffer each group (*N*=5). Each group was assigned a different label according to different concentrations of baculovirus 10^8^, 10^9^, and 10^10^ PFU administered subcutaneously. The first injection was done after mixing the purified virus with complete Freund’s adjuvant (Sigma-Aldrich, USA) except for the 10^10^ PFU dose. After 2 weeks after the first dose, the booster doses with the same concentrations were injected by mixing with incomplete Freund’s adjuvant (Sigma-Aldrich, USA). At the end of the month, the blood was harvested from the retro-orbital vein after local anesthesia. The anti-sera were screened by dot blot and western blot to confirm the display.

### Dot blot and western blot

Dot blot and western blot were performed using the Amersham™ ECL Western Blotting and analysis system (GE Healthcare, Buckinghamshire UK). All reagents were prepared and adapted in 24-well plates to include 200μl of any reagent or antibody. First, 0.2μm nitrocellulose was cut into small circles in a 24-well plate and then incubated for 15min with 3μl of AcRBD-sfGFP-64 and AcEGFP (~3×10^8^ PFU). All wells were blocked by soaking in 5% non-fat dry milk in PBS-T (PBS with Tween 20 0.1%) for 30 min at RT and washed twice with PBS-T. Primary antibodies (serum) were diluted 1:100 by dissolving in PBS-T and incubated for 30 min at RT before the membranes were washed thoroughly three times with PBS-T (3 × 5 min) followed by incubation with secondary antibody conjugated with HRP (1:1000) for 30 min at RT and washed three times with PBS-T (15 min × 1, 5 min × 2), and once with PBS (5 min). Quickly, the detection reagent was added and the membrane was examined by ChemiDoc XRS+ (Bio-Rad, USA). In the positive control of the dot blot, the anti-gp64 antibody was used [AcV5] (Abcam, USA). For western blot, the blotting step was done on a nitrocellulose membrane (0.2μm) following the instructions of the Bio-Rad blotting protocol that took 2.5 h in 20V. The detection was conducted similar to the dot blot in which, the serum from 10^10^ doses of AcRBD-sfGFP-V was used as the primary antibody and diluted at 1:100.

### Plaque reduction neutralization test (PRNT)

This experiment was conducted at Nawah Scientific Co. (Al-Asmarat, Egypt) following the PRNT protocol described before [45]. The serum sample was 10-fold serially diluted and mixed with hCoV-19/Egypt/NRC-03/2020 (Accession number on GSAID: EPI_ISL_430820) and then incubated along with the control untreated virus. Vero E6 was seeded in supplemented DMEM media and incubated for 24 h at 37 °C with 5% CO_2_. After the removal of culture media, 100μl of the sample was incubated with the cells for 1hr at 37°C followed by adding 3ml DMEM media and 2% agarose overlay and left to solidify before incubation at 37°C with 5% CO_2_. After 4 days, the plaques appeared and then a 10 % formalin was added for 2 h and stained with 0.1% crystal violet, and plaques were recorded as % inhibition = viral count (un-treated) - viral count (treated)/viral count (untreated) × 100.

### Estimation of the levels of IL-2, IL-4, IL-10, and IFN-γ in isolated splenocytes

Spleens were isolated from dissected mice and homogenized into a single-cell suspension using PBS buffer, then filtered with 40-μm Falcon™ Cell Strainers (fisher scientific, USA). The red blood cells were lysed by ammonium-chloride-potassium (ACK) lysing buffer (ThermoFisher, USA), followed by centrifugation for 5 min at 2000 RPM and removal of the supernatant. The cell pellets were washed with 100 μl of ACK buffer 2–3 times to get a clear white pellet; finally, the cells were preserved in RPMI media supplemented with 10 % FBS and 10 % DMSO at −80°C. The cells were thawed and then seeded overnight in 60mm plates and harvested for total protein extraction using cOmplete™ Lysis-M (Roche, Germany) following its protocol. IL-2, IFN-γ, IL-4, and IL-10 levels were measured using ELISA kits following the manufacturer’s instructions (Elabscience Biotechnology Inc, USA).

### Data analysis

Pairwise *t* test was used to evaluate the significance of the cytokines ELISA readings performed with GraphPad Prism 8.0 (GraphPad Software, Inc., San Diego, CA).

## Results

### Production of pseudotyped baculovirus displaying SARS CoV-2 RBD fused with sfGFP

**Fig. 1:**
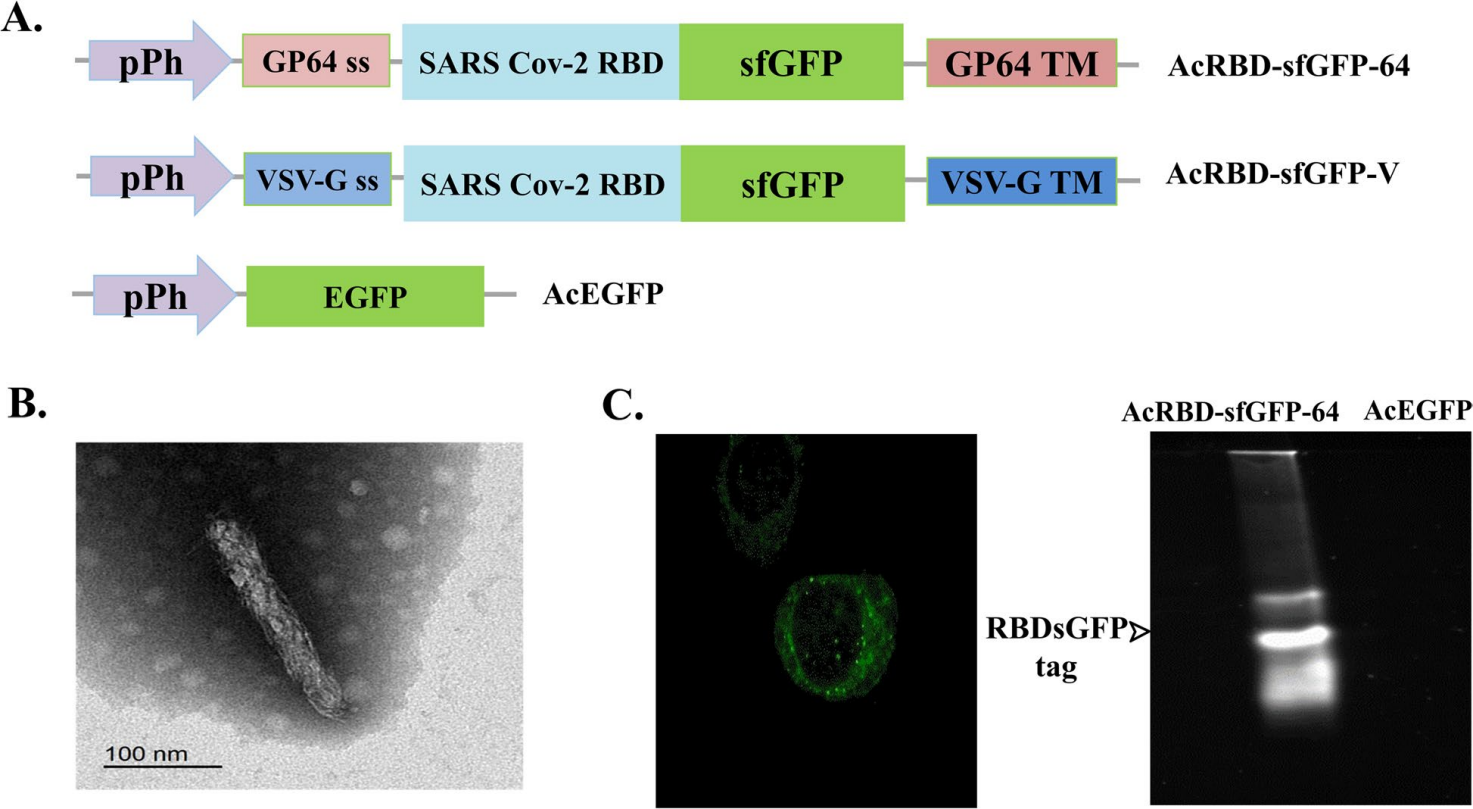
Production of pseudotyped baculovirus displaying the RBD-sfGFP based on gp64 and VSV G-proteins. **a** Schematic map for bacmids constructs; all constructs were under polyhedrin promoter (Pph). **b** TEM of AcRBD-sfGFP-64 showing a rod-shaped budded virus (BV) 30–70 nm in diameter and 200–400 nm in length. **c** Validation of the function of the displayed protein by showing fluorescence on Sf9 cell periphery and ER using fluorescence microscopy under 60× objective and with in-gel fluorescence

The signal sequence, TM, and CTD of Gp64 and VSV-G were used to display RBD-sfGFP on baculovirus; viruses were named AcRBD-sfGFP-64 and AcRBD-sfGFP-V, respectively (Fig. 1a). To confirm the morphology of the produced virus, AcRBD-sfGFP-64 was validated by TEM, which demonstrated an intact rod shape structure of the budded virus (BV) 30–70nm in diameter and 200–400 nm in length (Fig. 1b). The main aim of sfGFP incorporation is to localize the RBD hence the fluorescence image of the infected Sf9 cells with AcRBD-sfGFP-64 showed fluorescence foci, and around the periphery of the cell, which is an expected pattern for transmembrane proteins (Fig. 1c). In addition, the in-gel fluorescence of AcRBD-sfGFP-64 showed fluorescent bands indicating that the RBD-sfGFP is part of the virus in comparison to the control virus AcEGFP, which showed no fluorescent bands (Fig. 1c). Viruses were purified by anion exchange chromatography and viruses’ titers after endpoint dilution was 1.47×10^11^, 1.23×10^11^, and 1 × 10^11^ Pfu/ml for AcRBD-sfGFP-64, AcRBD-sfGFP-V, and AcEGFP, respectively.

### Screening of mice Sera against anti-RBD polyclonal antibodies

**Fig. 2:**
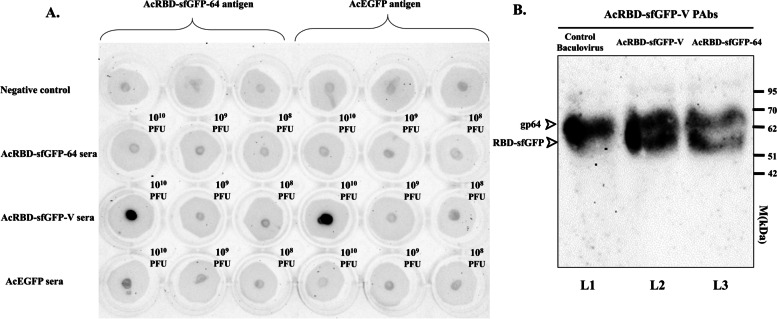
Production of polyclonal antibody by the pseudotyped virus from immunized BALB/C mice. **a** The virus displaying RBD produced antibodies that can bind both RBD and gp64 protein at 10^10^ pfu/ml in a serum sample obtained from mice injected with AcRBD-sfGFP-V subcutaneously without any adjuvant. **b** Western blot of the AcRBD-sfGFP-64, AcRBD-sfGFP-V, and the AcDH10Bac detecting differences between the RBD-sfGFP and gp64 bands. Two distinguished bands corresponding to gp64 (~67 kDa) and RBD-sfGFP (~ 51 kDa) were observed in both AcRBD-sfGFP-64 and AcRBD-sfGFP-V samples but only a single band corresponding to gp64 was observed in the AcDH10Bac

Our aim in this experiment was to prove the capability of the monomeric RBD to be recognized by and trigger the immune system and to optimize the dose of baculovirus injection as many previous studies used inconsistent doses. Mice were in good health during the 30-day experiment without mortality and the sera collected were preserved at −20°C until used. AcRBD-sfGFP-64, AcRBD-sfGFP-V, and AcEGFP sera of different injection doses; 10^8^, 10^9^, and 10^10^ PFU were tested by dot blot in which the antigens either AcEGFP or AcRBD-sfGFP-64 were blotted on membranes as seen in (Fig. 2A). The antibody that can optimally bind both AcEGFP and AcRBD-sfGFP-64 was detected at 10^10^ PFU of AcRBD-sfGFP-V subcutaneous serum without any adjuvant (Fig. 2A). However, the dot blot method could not distinguish whether the polyclonal antibodies in the serum were specific to the RBD or not. Therefore, western blot analysis was performed for AcRBD-sfGFP-64, AcRBD-sfGFP-V, and the AcDH10Bac to differentiate the RBD-sfGFP specific band from other bands that can be targeted by the polyclonal antibodies. Since Gp64 is the major envelope glycoprotein of AcMNPV BV, it is expected to be seen at around the 60–72 kDa band. Moreover, the fused RBD-sfGFP protein size was estimated to be 51 kDa using the protein molecular weight software tool (Bioinformatics.org). Interestingly, only two distinguished bands corresponding to gp64 around the size of 62-70 kDa and to RBD-sfGFP around 51-62 kDa for AcRBD-sfGFP-64 and AcRBD-sfGFP-V samples were shown, while only a single band corresponding to gp64 was shown for the AcDH10Bac (Fig. 2b). Even though, the AcMNPV BV envelope contains other proteins than gp64; they were not detected. Furthermore, the intensity of the RBD-sfGFP band of the AcRBD-sfGFP-V sample was more intense than the AcRBD-sfGFP-64 sample; the same was observed with the gp64 band (Fig. 2b L 2 & 3). Interestingly, the gp64 band was similar in both AcRBD-sfGFP-V and AcDH10Bac but decreased in AcRBD-sfGFP-64 (Fig. 2b).

### AcRBD-sfGFP-V anti-sera inhibits an Egyptian wild-type isolate of SARS CoV-2 measured by PRNT

**Fig. 3:**
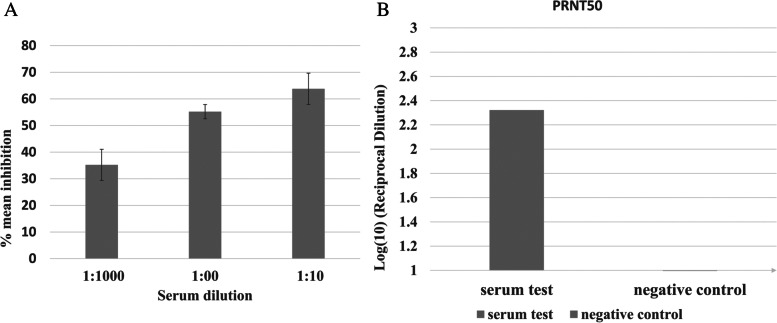
PRNT assay of the polyclonal antibodies produced by AcRBD-sfGFP-V immunization in BALB/C mice. **A** The bar chart shows the percent viral inhibition after 10-fold serial dilution. **B** A nonlinear relation is blotted and giving the equation *y* = −5.6833×^2^ + 8.45× + 61.033 where *y* substituted with 50 representing the neutralization titer (PRNT_50_) of SARS CoV-2 in Vero cells with PRNT_50_ > 100 shown in the bar chart with respect to the negative control (no serum)

This test is meant to examine the immunogenic ability of the produced PVs to produce mouse antibodies that can elicit neutralizing capability against the SARS-CoV-2 wild type. The obtained serum from mice injected with 10^10^ PFU of AcRBD-sfGFP-V was 10-fold serial dilution of 1:10, 1:100, and 1:1000. The results revealed that mice serum could reduce SARS-CoV-2 reproduction by approximately 30% at 1:1000, 50% at 1:100, and 70% at 1:10 (S2; Table. 2). Taking Log_10_ of the dilution reciprocal, the values 1, 2, and 3 represent 1:10, 1:100, and, 1:1000 respectively. Neutralization titer was determined between 100 and 200 as defined by the highest dilution that gives 50% inhibition (Fig. 3A, B).

### AcRBD-sfGFP-V and AcRBD-sfGFP-64 increase the levels of IL-2 and IFN-γ and decrease the levels of IL-4 and IL-10 in immunized mice

The preserved splenocytes were thawed and left overnight to attach; the cells count was between 24×10^6^–45×10^6^, and the viability was >90%. The total protein extracted from the splenocytes was analyzed by ELISA (*N*=3). The mean concentrations of IL-2 are 60.83 (SD=1.44), 83.75 (SD=2.5), and 76.25 (1.25) for AcEGFP, AcRBD-sfGFP-V, and AcRBD-sfGFP-64, respectively. In parallel, INF gamma mean titers were 574.6 (SD=5), 615.3 (SD=6.1), 646.6 (SD=8) for AcEGFP, AcRBD-sfGFP-V, and AcRBD-sfGFP-64, respectively. On the other hand, the mean values for IL-4 were 49.7 (SD=0.95), 35.9 (SD=1.23), 36.5 (SD=0.66), and IL-10 83.8(SD=5.3), 34.6 (SD=4.6), and 34.7 (SD=3.1) for AcEGFP, AcRBD-sfGFP-V, and AcRBD-sfGFP-64, respectively.

**Fig. 4:**
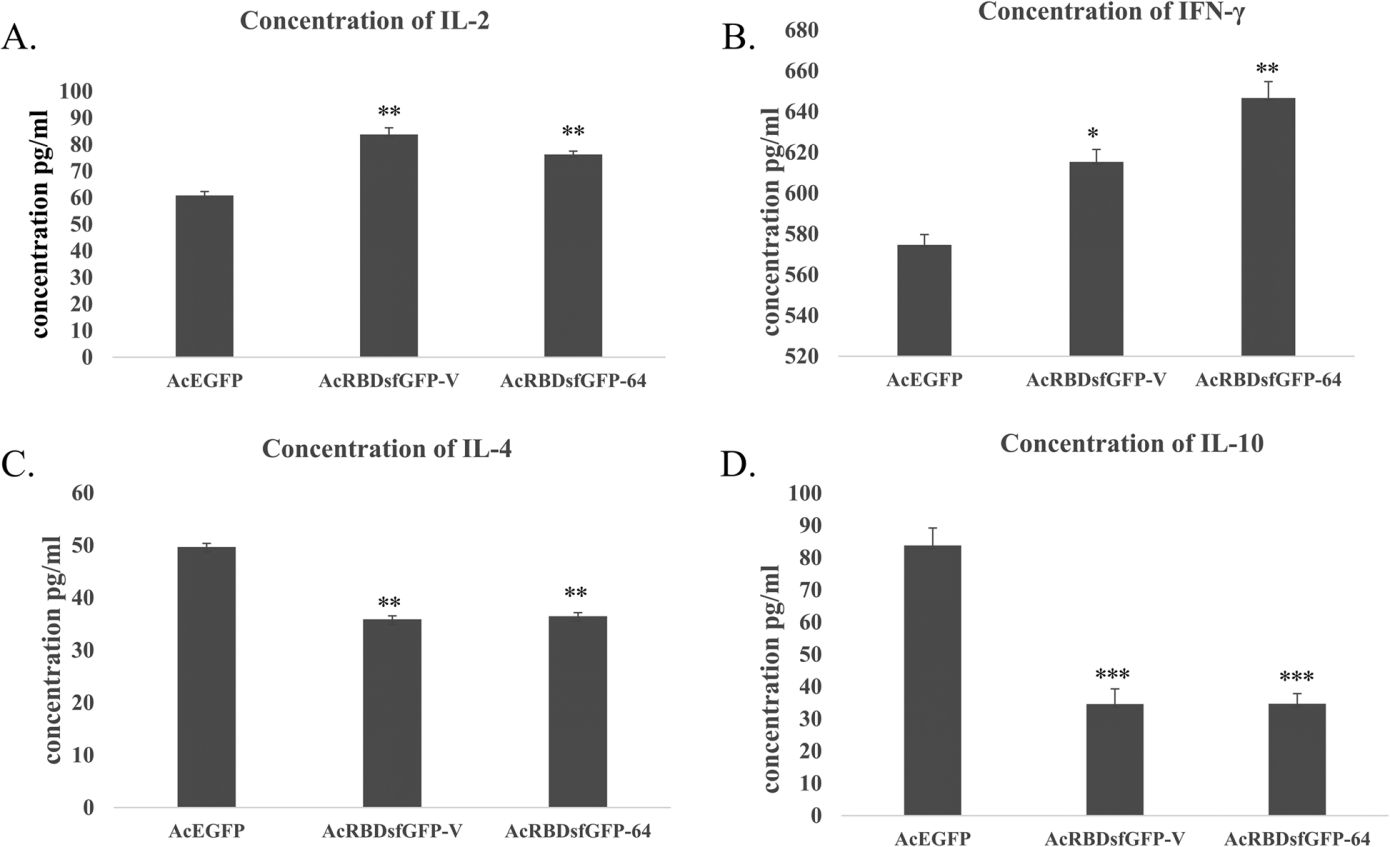
ELISA readings for splenic extracted cytokines show Th1 response in baculovirus displaying RBDsfGFP. **A** IL-2, **B** IFN-γ, **C** IL-4, and **D** IL-10 in splenocytes of mice after injection with AcEGFP, AcRBDsfGFP-V, and AcRBDsfGFP-64. Data presented as mean ± SD. *P value <0.05, **P value < 0.01, and ***P value < 0.001

The results showed a significant increase (*p*<0.01) in the levels of IL-2 and IFN-γ and a significant decrease (*p*<0.01) and (*p*<0.001) in IL-4 and IL10, respectively, of the isolated splenocytes from the immunized mice with AcRBD-sfGFP-V and AcRBD-sfGFP-64 compared to AcEGFP as shown in Fig. 4.

## Discussion

One of the main challenges with pseudotyping is the balance between increasing membrane incorporation of the target protein and maintaining a high titer of the virus. This point was addressed carefully in our work. Usually, the choice of the promoter, the type of the fusion partner, and the nature of the protein structure dictate the efficiency of protein display. Even though early promoters of baculovirus are preferred for pseudotyping, the strong, very late polyhedrin promoter is highly efficient too [10]. Moreover, the distribution of the displayed protein is a crucial factor for the pseudotyped virus characteristics as it determines the binding affinity for cellular receptors. Previous work on baculovirus displayed a target protein with VSV-G (TM+CTD) in comparison with gp64 (TM+CTD). It was reported that from 2 to 7 displayed molecules mediated by VSV-G can be distributed symmetrically around the virus surface, but gp64 only presents fewer molecules on the apical ends when examined using the immunogold labeling technique [46]. Such a strategy was reported in baculovirus displaying SARS-CoV spike ectodomain successfully by VSV-G (TM+CTD) [47]. RBDsfGFP fusion will enable us to track the protein in the infected cell and detect it using in-gel fluorescence and quantified by endpoint dilution. In contrast to GFP, sfGFP can tag membrane proteins to keep high-quality fluorescence [48, 49]. The results of in-gel fluorescence and endpoint dilution suggested that the fused RBD-sfGFP protein was efficiently displayed and did not negatively affect viral replication (Fig. 1c). Similarly, rabies virus envelope glycoprotein G was fused with the red fluorescence protein RFP and incorporated into the membrane to track virus particles in the entry process [50]. It is worth noting that purifying the viruses with the membrane ion-exchange chromatography method was efficient and did not alter the function of the displayed proteins according to the downstream experiments. The membrane ion-exchange chromatography method was done before; however, it was not tested for its impact on the displayed proteins with baculovirus [51].

The dot blot results confirmed that the VSV-G display is more efficient to trigger the immune response of BALB/C mice as seen in the intense bands of the concentration 10^10^ PFU in contrast to the display using gp64 with the same dose (Fig. 2a). Moreover, the absence of gp64 strong signal may be explained by the decrease of the distribution of molecules in gp64 (TM+CTD) in contrast to VSV-G (TM+CTD) as mentioned before. In addition, western blot showed two intense bands around 64 and 51 kDa with the AcRBD-sGFP-V sample; the bands were more intense than with the AcRBD-sGFP-64 sample when tested with the serum from mice injected with AcRBD-sfGFP-V. Interestingly, in the control baculovirus lane, the intensity of the band corresponding to gp64 was nearly the same as with the AcRBD-sGFP-V lane. This may indicate that the numbers of the displayed protein mediated by VSV-G are more abundant than the ones mediated by gp64. In another work, the results of the western blot in baculovirus displaying SARS CoV-2 spike observed a drastic reduction when displayed by gp64 (TM+CTD) compared to VSV-G (TM+CTD); however, degradation products were observed with the displayed full spike and S1 subunit but not with the RBD alone probably due to the presence of specific protease [52]. Hence, it may be advisable to display smaller domains of the spike such as the RBD on baculovirus to prevent such modifications on the structure. Usually, the dose range 10^8^–10^10^ PFU is recommended for eliciting the immune system as previously reported, which was within the range of our results [53–55]. Such different doses will depend on multi-factors such as the animal model, injection route, and adjuvant used. We have shown that a high dose of 10^10^ can be enough to trigger the immune system without an adjuvant. This can be of great value to the economics of vaccines since the supply chain around the world has been affected especially in developing countries. The sole appearance of Gp64, in addition to RBD, can be due to gp64 nature as it is the most abundant envelope protein. While the baculovirus BV envelope contains six more proteins other than gp64 [56]. These proteins are GP37 (Ac64), ODV-E25 (Ac94), ODV-E18 (Ac143), and BV/ODV-E26 (Ac16), F-like protein (Ac23), v-Ubi (Ac35) [55].

PRNT assay of the wild-type virus is a standard method indicating the efficacy of viral inhibition by neutralizing antibodies [57–59]. The value of PRNT50 is indicative of the strength of inhibition at a higher dilution. Our results show a PRNT50 value >100 after BALB/C immunization with 10^10^ PFU of AcRBD-sfGFP-V injected subcutaneously (Fig. 3). The result was comparable to what was reported using baculovirus displaying Japanese encephalitis virus E glycoprotein with (PRNT50 = 1:115.2) [60]. Microneutralization assay of baculovirus displaying VP1 of Human Enterovirus 71 gave 1:32 to 1:64; however, mice were injected subcutaneously with a lower dose of 108 PFU but with Freund’s adjuvant [54]. The display of Zika virus envelope protein on baculovirus with Freund’s adjuvant after three doses gave PRNT50 = 1:47.76 after 15μg/dose intraperitoneal injection in mice [61]. These data indicate that our system can elicit comparable neutralizing antibodies compared to other displayed proteins on baculovirus displayed antigen. Interestingly, baculovirus expressed the full spike and gave high neutralizing titer while the S1 and RBD did not show significant neutralization [62]. Moreover, the Ad5 viral vector expressing SARS-CoV-2-S1 protein showed a microneutralization titer (NT90) value of about <40 [63]. Quite similar values were also reported in another viral vector vaccine with a lower dose and immunization period and both with a single dose injection [64]. Finally, it seems that the use of the trimeric or monomeric form of the recombinant vaccine is a matter of debate since the findings agree on the efficacy of monomeric and trimeric forms [65, 66].

The IL-2 and IFN-γ were used as markers for the Th1 response shift. Both AcRBD-sfGFP-V and AcRBD-sfGFP-64 significantly increased IL-2 and IFN-γ; hence, this suggests that the displayed RBD induced Th1 response. Reduction in the concentration of IL-4 and IL-10 can result from the increased amount of INF- γ interfering with IL-4 gene expression [67]. It is worth noting that Th1 response was achieved in several RBD-based vaccines as demonstrated in (Table 1). For instance, the addition of 3M052 adjuvant to the RBD subunit vaccine is suggested to induce dendritic cells to stimulate CD8+T cells specifically. It was reported that antibodies against the RBD did not show antibody-dependent enhancement (ADE) in vitro. For the current work, we confirmed some of the pros of using the RBD such as induction of the Th1 response, which was reported with several vaccine platforms instead of vaccines that induced Th2 biased CD4+ T cell responses that aggravate respiratory diseases as reported before [32–39].

**Table 1:** RBD-based vaccines with different formulations and the type of immune response that they trigger

Vaccine name	Developer	Dose timing	Clinical trials	Vaccine type	Used adjuvant	Immune response mechanism	Th1/Th2 immune balance	Reference
ArCov	Chinese Academy of Military Medical Sciences	0 and 28 days (100 μg)	Phase 1 clinical trial (ChiCTR2000034112)	mRNA encodes the SARS-CoV-2 RBD	Lipid nanoparticle encapsulated mRNA (mRNA-LNP)	Increase the level of CD4^+^ and CD8^+^ effector memory T, IFN-γ, TNF-α, and IL-2 in isolated splenocytes	Induce Th1 cellular immune response	[32]
BNT162b1	BioNTech and Pfizers	0 and 28 days (1–50 μg)	Phase 1 and 2 clinical trial	mRNA encodes the SARS-CoV-2 RBD	Lipid nanoparticle encapsulated mRNA (mRNA-LNP)	Elevation of CD4^+^ and CD8^+^ T cell responses and increase in the secretion of IFN-γ, IL-2, and IL-12p70	Induce Th1 cellular immune response	[33]
Folded RBD-PreS fusion vaccine	-	Three times in 3 weeks (20 or 40 μg)	-	SARS-CoV-2 RBD fused to the N- and C-terminus of hepatitis B virus (HBV) surface antigen PreS expressed in *E. coli* expression system	Aluminum hydroxide	Induce production of allergen-specific IgG responses (IgG1 and IgG 4)	-	[34]
ZF2001	Institute of Microbiology, Chinese Academy of Sciences, and Anhui Zhifei Longcom Biopharmaceutical	0, 30, and 60 days (25 μg per dose)	Phase 3 clinical trial	Adjuvant-based protein subunit vaccine	Aluminum hydroxide	Elevation of the levels of MHC class II-related genes, including cd74 and H2-Aa and induced the presentation of antigens to CD4^+^ T cells via MHCII molecules	Increase TH1 cytokine production (IFN-γ, IL-2) and TH2 cytokine production (IL-4) cytokine production resulting in Th1/Th2 balanced cytokines	[35, 36]
A yeast-expressed RBD-based SARS-CoV-2 vaccine	-	0, 4, and 9 weeks	-	Yeast expression system	3M-052-aluminum	Increase nAbs & elevation of CD4^+^ and CD8^+^ T cell responses	Induce Th1-biased CD4^+^ T cell reactions	[37]
Unnamed recombinA2:I8accine	West China Hospital	0, 21 days and 0, 14, 28 days	Phase 2 clinical trial	The baculovirus expression system	Aluminum hydroxide gel	Increased levels of IgM and IgG in sera and increased the levels of IFN-γ and IL-4 from isolated lymphocytes	Enhanced and TH1/TH2 balanced cytokine production	[38]
Mutant RBD vaccines	-	0 and 14 days	-	HIV-1 backbone	Aluminum	High geometric mean titers (GMTs) of neutralizing antibodies (nAbs)	-	[39]

## Conclusions

In this study, we confirmed that SARS CoV-2 pseudotyped baculovirus can effectively display the RBD and maintain functionality by producing neutralizing antibodies against SARS CoV-2 local isolate in Egypt (hCoV-19/Egypt/NRC-03/2020). Our preliminary data support that baculovirus-based RBD display could be a safe and effective approach as a vaccine. In addition, dose 1010 PFU without adjuvant is highly recommended due to its safety and efficacy in BALB/C mice. Such results are very promising for further investigations into display-based vaccines. Indeed, we faced some limitations such as inaccessibility to animals for a challenging study and overall biosafety level three and inability to test new variants as well. However, we were interested to study the baculovirus as a platform for RBD display with emphasis on its adjuvant effect, safety, and Th1/Th2 response balance. Overall, the model can help immunologists to study its efficacy as a safe vaccine.

## Supplementary Information

**Additional file 1: Table S1.** Vaccines are adopted in several systems and have different immune responses.

**Additional file 2: Table S2.** PRNT results indicated by viral inhibition percentage in three 10-fold dilutions. The 10-fold serially diluted serum shows a decrease in SARS-CoV-2 reproduction in Vero cells with a minimum of 30% inhibition after 1:1000 serum dilution and more than 71% after 1:10 serum dilution.

**Additional file 3.** Sequencing results of the clone AcRBD-sfGFP-V. >pFastDualvsvgTMSS RPDGFP_pFASTBAC-R-5HD372-11108153806.

**Additional file 4.** S4_ ELISA plate reader raw dataR1.

## Data Availability

Not applicable.

## Abbreviations

SARS CoV-2: Severe acute respiratory syndrome coronavirus 2
RBD: Receptor-binding domain
PRNT: Plaque reduction neutralization test
GP64: Glycoprotein 64
VSV-G: Vesicular stomatitis virus glycoprotein G
AcMNPV: *Autographa californica* multiple nucleopolyhedrovirus
NT90: Neutralization titer 90
NT50: Neutralization titer 50
PFU: Particle-forming unit
TCID: Tissue culture infectious dose
MOI: Multiplicity of infection
sfGFP: Superfolder green fluorescent protein
MD: Mature domain
CTD: C-terminal domain
TM: Transmembrane
