# Supplementary material for: Baculovirus displaying SARS-CoV-2 spike RBD promotes neutralizing antibody production in a mouse model
Source: J Genet Eng Biotechnol. 2023 Feb 9;21:16. doi: 10.1186/s43141-023-00472-2 (PMC9910779; doi:10.1186/s43141-023-00472-2)
Supplement: Supplementary file 2 — Additional file 2: Table S2. PRNT results indicated by viral inhibition percentage in three 10-fold dilutions. The 10-fold serially diluted serum shows a decrease in SARS-CoV-2 reproduction in Vero cells with a minimum of 30% inhibition after 1:1000 serum dilution and more than 71% after 1:10 serum dilution. [file 43141_2023_472_MOESM2_ESM.docx]

Table.2. PRNT results indicated by viral inhibition percentage in three 10-fold dilutions. The 10-fold serially diluted serum shows a decrease in SARS-CoV-2 reproduction in Vero cells with minimum 30% inhibition after 1:1000 serum dilution and more than 71% after 1:10 serum dilution.

| Replica | Dilution | Virus Control  (PFU/ml) | Viral Titer  Post-Treatment (PFU/ml) | Viral Inhibition (%) |
| --- | --- | --- | --- | --- |
| **Replicate_1** | **1:10** | ***3.5 * 10^5^*** | ***1.0 * 10^5^*** | ***71.4 %*** |
|  | **1:100** |  | ***1.5 * 10^5^*** | ***57.1 %*** |
|  | **1:1000** |  | ***2.5 * 10^5^*** | ***28.6 %*** |
| **Replicate_2** | **1:10** | ***3.5 * 10^5^*** | ***1.3 * 10^5^*** | ***62.9 %*** |
|  | **1:100** |  | ***1.7 * 10^5^*** | ***51.4 %*** |
|  | **1:1000** |  | ***2.3 * 10^5^*** | ***34.3 %*** |
| **Replicate_3** | **1:10** | ***3.5 * 10^5^*** | ***1.5 * 10^5^*** | ***57.1 %*** |
|  | **1:100** |  | ***1.5 * 10^5^*** | ***57.1 %*** |
|  | **1:1000** |  | ***2.0 * 10^5^*** | ***42.8 %*** |
