# Supplementary material for: Baculovirus displaying SARS-CoV-2 spike RBD promotes neutralizing antibody production in a mouse model
Source: J Genet Eng Biotechnol. 2023 Feb 9;21:16. doi: 10.1186/s43141-023-00472-2 (PMC9910779; doi:10.1186/s43141-023-00472-2)
Supplement: Supplementary file 3 — Additional file 3. Sequencing results of the clone AcRBD-sfGFP-V. >pFastDualvsvgTMSS RPDGFP_pFASTBAC-R-5HD372-11108153806. [file 43141_2023_472_MOESM3_ESM.docx]

Sequencing results of the clone AcRBD-sfGFP-V

>pFastDual-VSV-TMSS v7_pFASTBAC-R-5HD370-11108153806

TGGACTTCTCGAACAAGCTTTTACTTTCCAAGTCGGTTCATCTCTATGTCTGTATAAATCTGTCTTTTCTTGGTGTGCTTTAATTTAATGCAAAGATGGATACCAACTCGGAGAACCAAGAATAGTCCAATGATTAACCCTATGATAAAGAAAAAAGAGGCAATAGAGCTTTTCCAACTACTGAACCAACCTCTAGATTCGAAAGCGGCCGCGACTAGTGAGCTCGTCGACGTAGGCCTTTGAATTCGCAATTCACCCCAATGAATAAAAAGGCTAAGTACAAAAGGCACTTCATGGATCCGCGCCCGATGGTGGGACGGTATGAATAATCCGGAATATTTATAGGTTTTTTTATTACAAAACTGTTACGAAAACAGTAAAATACTTATTTATTTGCGAGATGGTTATCATTTTAATTATCTCCATGATCTATTAATATTCCGGAGTATACGGACCTTTAATTCAACCCAACACAATATATTATAGTTAAATAAGAATTATTATCAAATCATTTGTATATTAATTAAAATACTATACTGTAAATTACATTTTATTTACAATCACTCGACGAAGACTTGATCACCCGGGATCTCGAGCCATGGTGCTAGCAGCTGATGCATAGCATGCGGTACCGGGAGATGGGGGAGGCTAACTGAAACACGGAAGGAGACAATACCGGAAGGAACCCGCGCTATGACGGCAATAAAAAGACAGAATAAAACGCACGGGTGTTGGGTCGTTTGTTCATAAACGCGGGGTTCGGTCCCAGGGCTGGCACTCTGTCGATACCCCACCGAGACCCCATTGGGACCAATACGCCCGCGTTTCTTCCTTTTCCCCACCCCAACCCCCAAGTTCGGGTGAAGGCCCAGGGCTCGCAGCCAACGTCGGGGCGGCAAGCCCTGCCATAGCCACTACGGGTACGTAGCCAACCACTAGAACTATAGCTAGAGTCCTGGGCGAACAAACGATGCTCGCCTTCCAGAAAACCGAGGATGCGAACCACTTCATCCGGGGTCAGCACCACCGGCAAGCGCCGCGACGGCCGAGGTCTACCGATCTCCTGAAGCAAGGGCAGATCCGTGCACAGCACCTTGCCGTAGAAGAACAGCAAGGCCGCCAATGCCTGACGATGCGTGGAGACCGAAACCTTGCGCTCGTTCGCCAGCCAGGACAGAAATGCCTCGACTTCGCTGCTGCCCAAGGTTGCCGGGTGACGC

Using the universal primer **pFASTBAC-R** the sequence revealed the **mature domain sequence** (transmembrane and cytoplasmic domain) being cloned in the right position between ***XbaI/HindIII*** without any mutation (highlighted in **yellow**). Also the **VSV-G** signal sequence was cloned between the ***BamHI/EcoRI (highlighted in red).***

>pFastDualvsvgTMSS RPDGFP_pFASTBAC-R-5HD372-11108153806

AACCGGATTCTCGACAAGCTTTTACTTTCCAAGTCGGTTCATCTCTATGTCTGTATAAATCTGTCTTTTCTTGGTGTGCTTTAATTTAATGCAAAGATGGATACCAACTCGGAGAACCAAGAATAGTCCAATGATTAACCCTATGATAAAGAAAAAAGAGGCAATAGAGCTTTTCCAACTACTGAACCAACCTCTAGATTTGTAGAGCTCATCCATGCCATGTGTAATCCCAGCAGCAGTTACAAACTCAAGAAGGACCATGTGGTCACGCTTTTCGTTGGGATCTTTCGAAAGGACAGATTGTGTCGACAGGTAATGGTTGTCTGGTAAAAGGACAGGGCCATCGCCAATTGGAGTATTTTGTTGATAATGGTCTGCTAGTTGAACGGAACCATCTTCAACGTTGTGGCGAATTTTGAAGTTAGCTTTGATTCCATTCTTTTGTTTGTCTGCCGTGATGTATACATTGTGTGAGTTAAAGTTGTACTCCAGTTTGTGTCCGAGAATGTTTCCATCTTCTTTAAAATCAATACCTTTTAACTCGATACGATTAACAAGGGTATCACCTTCAAACTTGACTTCAGCACGCGTCTTGTAGGTCCCGTCATCTTTGAAAGATATAGTGCGTTCCTGTACATAACCTTCGGGCATGGCACTCTTGAAAAAGTCATGCCGTTTCATGTGATCCGGATAACGGGAAAAGCATTGAACACCATAGGTCAGAGTAGTGACAAGTGTTGGCCATGGAACAGGTAGTTTTCCAGTAGTGCAAATAAATTTAAGGGTGAGTTTTCCGTTTGTAGCATCACCTTCACCCTCTCCACGGACAGAAAATTTGTGCCCATTAACATCACCATCTAATTCAACAAGAATTGGGACAACTCCAGTGAAAAGTTCTTCTCCTTTGGAACCACCACTACCGGACCCACCGGATCCCTTTTTGGGCCCACAAACTGTAGCCGGGGCGTGGAGGAGCTCAAAGGACAATACTACCACTCTATAAGGTTGAAACCCCACTCCGTTTGTTGGTTGAAAGCCATAAGATTGAAGAAGAAAG

After cloning the RBDsfGFP in the previous cloning vector pFastDual-VSV-TMSS, the plasmid was sent for sequencing. Using the universal primer **pFASTBAC-R** the sequence revealed the **mature domain sequence** (transmembrane and cytoplasmic domain) being cloned in the right position between ***XbaI/HindIII*** without any mutation (highlighted in **yellow**). Moreover, the RBDsfGFP was cloned successfully between ***XbaI/EcoRI (highlighted in green).***
